# An essential role for Ran GTPase in epithelial ovarian cancer cell survival

**DOI:** 10.1186/1476-4598-9-272

**Published:** 2010-10-13

**Authors:** Véronique Barrès, Véronique Ouellet, Julie Lafontaine, Patricia N Tonin, Diane M Provencher, Anne-Marie Mes-Masson

**Affiliations:** 1 Centre de recherche du Centre hospitalier de l'Université de Montréal (CRCHUM)/Institut du cancer de Montréal,1560 Sherbrooke Est, Montreal, H2L 4M1, Canada; 2 Department of Medicine, McGill University, Montreal, Canada; 3 Department of Human Genetics, McGill University, Montreal, Canada; 4 Research Institute of McGill University Health Centre (RI-MUHC), 1650 Cedar Avenue, Montreal, H3G 1A4, Quebec, Canada; 5 Department of Obstetric-Gynecology/Université de Montréal, Montreal, Canada; 6 Départment of Medecine, Université de Montréal, Montreal, Canada

## Abstract

**Background:**

We previously identified that Ran protein, a member of the Ras GTPase family, is highly expressed in high grade and high stage serous epithelial ovarian cancers, and that its overexpression is associated with a poor prognosis. Ran is known to contribute to both nucleocytoplasmic transport and cell cycle progression, but its role in ovarian cancer is not well defined.

**Results:**

Using a lentivirus-based tetracycline-inducible shRNA approach, we show that downregulation of Ran expression in aggressive ovarian cancer cell lines affects cellular proliferation by inducing a caspase-3 associated apoptosis. Using a xenograft tumor assay, we demonstrate that depletion of Ran results in decreased tumorigenesis, and eventual tumor formation is associated with tumor cells that express Ran protein.

**Conclusion:**

Our results suggest a role for Ran in ovarian cancer cell survival and tumorigenicity and suggest that this critical GTPase may be suitable as a therapeutic target.

## Background

Ovarian cancer is the leading cause of death from gynecological cancer in western countries [1,2]. Being largely asymptomatic, more than 70% of patients are diagnosed with advanced stage disease. Despite various modifications in ovarian cancer therapy, there has been very little progress in overall patient survival for the past 30 years and screening programs to detect early disease have not been successful to date [3]. Approximately 90% of ovarian cancers are of epithelial origin (EOC) and these tumors can be classified into different histopathologies, of which the serous histotype is the most common [4]. Low malignant potential serous tumors have a five year survival rate of 90-95%, whereas the survival rate for invasive serous cancers drops dramatically to 35-40% [3].

Using a molecular profiling analysis, we previously identified different genes that can distinguish between low malignant potential tumors and invasive EOC [5]. Among interesting candidates, we studied the expression of the Ras-related nuclear protein Ran [5] using an immunohistochemistry approach on an EOC serous tissue microarray. Ran overexpression was associated with higher tumor grade and advanced stage disease. Moreover, Ran was the most significant marker able to predict patient survival with the highest combination of sensitivity and specificity [6].

The Ran protein is a small GTPase of the Ras superfamily known to play different roles in normal cell physiology. One of its major functions is to regulate the nucleocytoplasmic transport of molecules through the nuclear pore complex [7,8]. It has been proposed that the unusual localization of oncogenes and/or tumor suppressor proteins can be affected by Ran signaling in different types of cancer [9]. Ran is also involved in cell cycle progression through the regulation of mitotic spindle formation [10]. Deregulation of this process may lead to genomic instability, which is common in EOC. Overexpression of Ran GTPase has also been observed in various other malignancies when compared to their normal tissue, including stomach, colon, pancreas, lung and kidney cancer [11-13]. These observations indicate that the deregulation of Ran expression may be an important event in cell transformation or cancer progression [11].

To better understand the role of the Ran GTPase in ovarian tumorigenesis, we investigated the effects of Ran depletion in two aggressive EOC cell lines. Here, we show that loss of Ran expression leads to cell death by a caspase-3 associated apoptosis *in vitro*. Downregulation of Ran *in vivo *was also associated with tumor regression in SCID mice. This study demonstrates that the expression of Ran is important for EOC cell survival and suggests that Ran may be a suitable therapeutic target for the treatment of ovarian cancers.

## Results

### Ran expression in transduced cells

**Figure 1:**
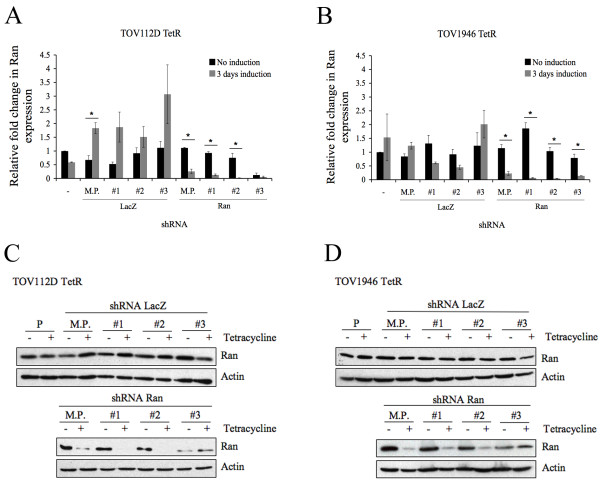
**Ran expression in parental cell lines, mixed populations and clones**. Comparison of Ran mRNA levels by Q-PCR in TOV112D TetR (a) and TOV1946 TetR (b) parental cell lines, shRNA Ran or LacZ mixed populations and clones. Levels were normalized to parental cell line without induction. Values represent the mean ± SD of two separate experiments each performed in duplicate. Western blot analysis of TOV112D TetR (c) and TOV1946 TetR (d) parental cell lines, mixed populations and clones shRNA Ran or LacZ without and after three days of induction. ß-actin was used as loading control. P: Parental cell line, M.P.: Mixed population, #1, #2 and #3: independent clones. Mann-Whitney test indicates a significant difference with a p-value < 0.05 (*).

We have previously shown that Ran GTPase is overexpressed in invasive serous EOC as compared to low malignant potential serous tumors [6]. To better understand the role of Ran in ovarian tumorigenesis, we downregulated its expression using a lentivirus-based inducible short-hairpin RNA (shRNA) strategy in two aggressive EOC cell lines derived in our laboratory (Figure 1) [14,15]. TOV112D is derived from a high grade endometrioid tumor and has been extensively characterized [15]. TOV1946 originates from a high grade serous carcinoma, which is the most common EOC histotype. Both cell lines harbor p53 mutations, the most common genetic lesion associated with high grade serous cancers. Transfection of pcDNA6/TR generated clonal derivates of these cell lines expressing the tetracycline repressor (TetR). This allows the inducible expression of the shRNA when tetracycline is added to the media, thus preventing basal target gene knockdown. After transduction of the pLenti-X1 Puro DEST vector containing the shRNA sequence specific to Ran or LacZ (as a control) in cells, we generated mixed populations and different independent clones of TOV112D TetR and TOV1946 TetR expressing shRNA Ran or LacZ. Q-PCR (Figure 1A and 1B), as well as immunoblot (Figure 1C and 1D) assays, showed decreased Ran expression in both mixed populations and clones expressing shRNA specific to Ran after induction. Despite some variation seen at mRNA and protein level in Ran expression after induction of the control shRNA LacZ (Figure 1), these minor modulations seem to be related to clonal effects and are not affecting the phenotype of the clones.

### Effects of Ran downregulation on cellular proliferation

**Figure 2:**
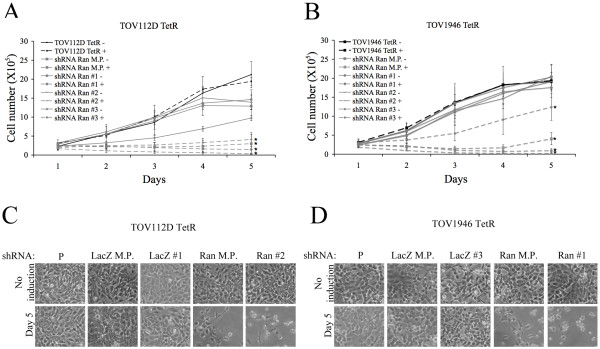
**Effect of Ran depletion on proliferation**. Growth curves for TOV112D TetR (a) and TOV1946 TetR (b) parental cell lines, mixed populations and clones expressing shRNA Ran. Cells were induced with tetracycline three days prior to day 0. Cells were trypsinized and counted every 24h for five days. Values represent mean ± SD of duplicate wells from three independent experiments. Cellular morphology of TOV112D TetR (c) and TOV1946 TetR (d) on day 5 of the growth curve. Loss of Ran GTPase expression results in cell number reduction and round shaped cells. All pictures were taken at 100× magnification. P: Parental cell line, M.P.: Mixed population, #1, #2 and #3: independent clones. Mann-Whitney test indicates a significant difference relative to the non-induced parental cell line with a p-value < 0.05 (*). There was no significant difference between the different non-induced cells.

We measured cellular proliferation of TOV112D TetR and TOV1946 TetR transduced with shRNA Ran in order to examine if cellular growth was affected by the loss of Ran. Cells expressing shRNA Ran or LacZ were induced with 1 μg/mL tetracycline three days prior to being seeded. We noted a significant decrease in cellular proliferation in both the mixed population and the clones with reduced Ran expression in comparison to their non-induced counterpart. We also noted that the reduction in cell proliferation may correlate with Ran expression levels. Tetracycline induction of clone shRNA Ran #3/TOV1946 TetR resulted in a partial reduction of Ran protein levels and the effect of this partial reduction on cell proliferation was modest when compared to clones 1 and 2, where Ran inhibition was stronger (Figure 2A and 2B). Induction of the shRNA LacZ did not affect cell proliferation (Additional file 1).

### Loss of Ran expression triggers apoptosis in EOC cells

**Figure 3:**
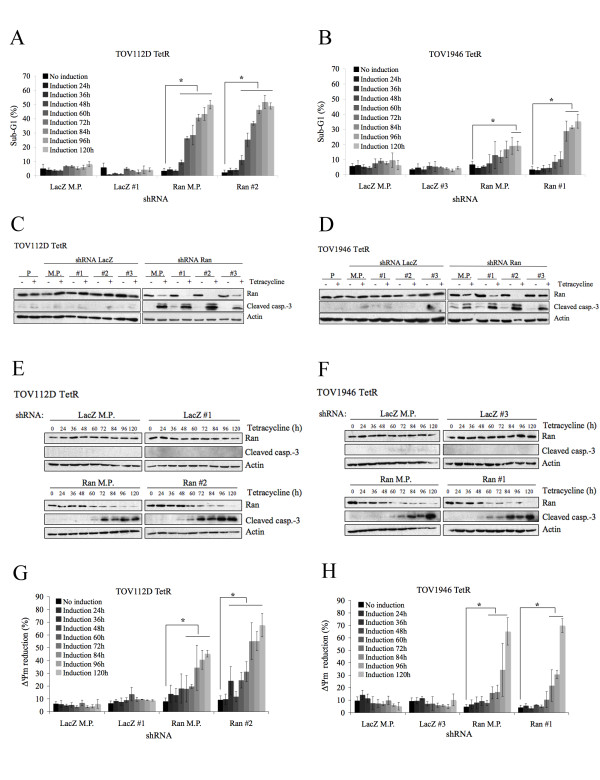
**Loss of Ran expression results in a caspase-3 associated apoptosis in ovarian cancer cell lines**. TOV112D TetR (a) and TOV1946 TetR (b) parental cell lines, mixed populations and clones expressing shRNA LacZ and Ran were induced with tetracycline for the indicated times and then fixed in 70% ethanol. Cells were treated with propidium iodide and sub-G_1 _peaks were quantified by flow cytometry. Values represent the mean ± SD of three independent experiments. Western blot analysis showing caspase-3 cleavage in cell depleted in Ran expression after 3 days of induction in TOV112D TetR (c) and TOV1946 TetR (d). Kinetic of caspase-3 cleavage by western blot analysis in TOV112D TetR (e) and TOV1946 TetR (f) parental cell lines, mixed populations and clones expressing shRNA LacZ and Ran. TOV112D TetR (g) and TOV1946 TetR (h) parental cell lines, mixed populations and clones expressing shRNA LacZ and Ran were induced with tetracycline for the indicated time and then treated with TMRE for 20 minutes. ΔΨm reduction was measured by flow cytometry. Values represent the mean ± SD of three independent experiments. P: Parental cell line, M.P.: Mixed population, #1, #2 and #3: independent clones. Mann-Whitney test indicates a significant difference relative to the non-induced sample with a p-value < 0.05 (*).

After induction of shRNA expression, cells depleted for Ran were rounder than observed with the parental cell line and tended to detach from the culture surface, a morphology characteristic of apoptotic cells (Figure 2C and 2D). Moreover, cell numbers actually decreased during shRNA Ran induction (Figure 2A and 2B). In order to determine if these cells were dying by apoptosis, we used propidium iodide (PI) to analyze the DNA content of the cells induced with tetracycline for different time periods and examined the sub-G_1 _fraction. Cells in sub-G_1 _phase represent hypodiploid DNA typically associated with late apoptosis or necrosis [16]. Since the different clones expressing shRNA Ran all behaved similarly, we subsequently looked at clones where Ran knockdown was most evident in addition to the mixed population. We observed a rapid sub-G_1 _accumulation after tetracycline induction in TOV112D TetR shRNA Ran, and a similar sub-G_1 _phase appeared, although more slowly, in TOV1946 TetR cells (Figure 3A and 3B). The proportion of sub-G_1 _fraction was comparable in mixed populations and clones, but was greater in the TOV112D TetR shRNA Ran compared to the TOV1946 TetR shRNA Ran. No increase in the sub-G_1 _fraction was observed at any time in the shRNA LacZ controls (Figure 3A and 3B).

Sub-G_1 _DNA is generated by late apoptotic or necrotic cells. To confirm that cells had undergone apoptosis and not necrosis following Ran depletion, we examined caspase-3 activation, a key downstream step in the apoptosis pathway [17]. After three days of induction with tetracycline, caspase-3 was cleaved in both mixed populations and clones expressing shRNA Ran in both cell lines (Figure 3C and 3D). We assayed caspase-3 cleavage under different induction time periods, and observed by western blotting that cleavage appeared after 60 h in both cell lines, and a slight band was apparent after 48 h induction in the shRNA Ran clones from both cell lines (Figure 3E and 3F). Therefore, the caspase-3 cleavage appears before the sub-G_1 _cells population in both cell lines. No cleavage of the caspase-3 was observed in the controls.

Mitochondria play a key role in the regulation of apoptosis with depolarization of the outer mitochondrial membrane being a point of no return in the apoptotic cascade [18,19]. Using tetramethylrhodamine ethylester (TMRE), we assayed outer membrane depolarization and noted that, in both cell lines, a decrease Ran expression resulted in a reduced outer membrane potential, observable after 48-60 h (Figure 3G and 3H). This membrane potential reduction was not seen in control shRNA LacZ. Thus, apoptosis was triggered between 48-60 h after Ran depletion in both cell lines, indicating that decrease of Ran expression promotes caspase-3 associated apoptosis.

### Knockdown of Ran causes regression of tumors in vivo

**Figure 4:**
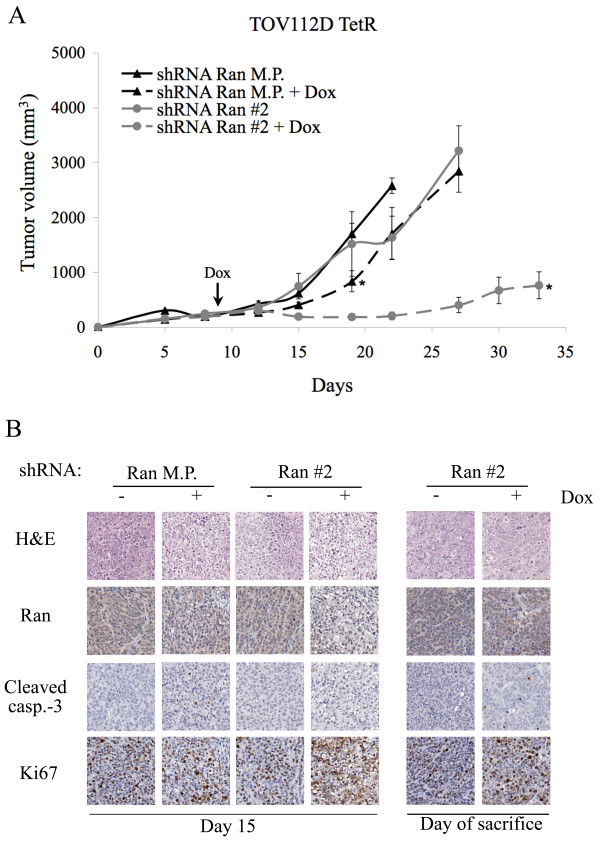
**Loss of Ran expression results in tumor regression *in vivo***. A. TOV112D TetR mixed population and clone expressing shRNA Ran were subcutaneously injected in SCID mice and tumor growth was measured biweekly. All mice developed tumors albeit of varying size and tumor volume. Doxycycline-supplemented food inducing shRNA expression started at day 9. M.P: Mixed population, Ran #2: clone. Values represent mean ± SE of groups of 4 mice. Mann-Whitney test indicates a significant p-value < 0.05 (*). B. Immunohistochemistry analysis of the xenografts on day 6 of the doxycycline-supplemented food showing morphological hematoxylin eosin (HE) staining and Ran, cleaved caspase-3 and Ki67 expression. Slides were obtained from contiguous tissues.

We showed that EOC cells are dependant on Ran expression to survive *in vitro*. In order to understand if Ran is also important for tumor cell survival *in vivo*, we used a xenograft-based model of tumor growth in SCID mice. As we have previously shown that TOV112D cells are highly aggressive when tested in a xenograft model [15], we chose to focus on this cell line for *in vivo *experiments. The short time course of tumor formation also minimized confounding effects related to the possible loss of shRNA expression in mice. After nine days, when tumors reached an average size of 150 mm^3^, mice were given food supplemented with doxycycline in order to induce shRNA expression in tumor cells. Loss of Ran expression, in tumors expressing the mixed population shRNA Ran, resulted in a modest delay in tumor growth, where the difference in tumor size was statistically significant at day 19 (Figure 4A). In mice injected with cells containing an inducible clone of shRNA Ran, tumor growth was delayed for several days after induction of shRNA expression and tumor sizes were statistically different at every measurement time point after day 15. Mice were sacrificed when tumors reached about 2300 mm^3^. All mice of the clone shRNA Ran non-induced group were sacrificed by day 27, while in the group where shRNA Ran was expressed, the last mouse was sacrificed at day 54. No major differences were observed in tumor volume between the induced and non-induced group of mice expressing shRNA LacZ in mixed populations or clones (Additional file 2). At day 0, 3 and 6 after shRNA induction and at the day of sacrifice, one mouse of each group was sacrificed to analyze tumor histology. The different stainings were performed on contiguous sections of the tissue microarray and the average and most representative staining were chosen for presentation. Analysis of Ran protein expression by immunohistochemistry demonstrated a reduced cytoplasmic expression in shRNA Ran mice after six days of induction with doxycycline-supplemented food (Figure 4B). In these tumors, we noted the presence of cleaved caspase-3 in the nucleus, which was absent in the controls. Ki67 staining showed that nuclear localization of the protein was impaired when Ran was downregulated (Figure 4B). Tumor analysis of the mice from the shRNA Ran/doxycycline-supplemented food group at the day of sacrifice showed that Ran levels were comparable to controls (Figure 4B). Despite the fact that tumors from shRNA LacZ mixed population grew faster than the clone, which is due to a clonal effect, no other differences were seen in shRNA LacZ with or without induction (Additional file 3). These results not only suggest that reduced expression of Ran is associated with tumor growth inhibition, but also suggest a strong selection pressure for Ran expression in those tumors that eventually develop in mice.

## Discussion

We previously demonstrated that Ran, a small GTP binding protein belonging to the Ras super-family, is overexpressed in invasive versus low malignant potential EOCs. Furthermore, the level of Ran overexpression correlates with a poorer prognosis among patients with high-grade invasive EOC [6]. Ran has previously been reported to be overexpressed by several other cancer types, such as stomach, colon, pancreas, lung and kidney cancer [11-13,20]. In this study we demonstrated, using a shRNA approach, that Ran appears to be essential for EOC cell survival and that knockdown of Ran expression results in the caspase-3 associated apoptosis of tumor cells.

We demonstrate here that downregulation of Ran not only affects cellular proliferation but also results in a decrease in the total number of cells. This result is in line with other studies showing that Ran depletion in cancer cells, using shRNA or siRNA, is associated with inhibition of cell growth and cell death [12,20].

Inhibition of Ran expression caused cells to undergo apoptosis as measured by cleaved caspase-3 protein levels and by FACS analysis. Ran plays several essential functions in cell physiology and it is not clear which of these functions might be responsible for these effects on tumor cell survival. One of Ran's GTPase-related functions is to regulate translocation of RNA and proteins through the nuclear pore complex, including AKT, p53, PTEN, NFκB and cyclins [7,9,21]. Ran also functions, independent of its role in nuclear pore transport, as a signaling molecule regulating microtubule polymerization during mitosis, deregulation of which may block cell cycle progression and lead to cell death. We thus analyzed the effect of Ran knockdown on cell cycle progression. As is generally observed in high grade serous cancers, both the TOV112D and TOV1946 cell lines harbor p53 mutations which correspond to those identified in the original tumor tissue [14,15]. Although one could speculate that this lesion may influence the response to loss of Ran expression, this seems unlikely since it has previously been shown that the effects of Ran loss on cell viability and hypodiploid DNA content are indistinguishable whether wild-type or isogenic cells deficient in p53 are used [12]. We did not observe cell cycle arrest in our clones in any phase of the cell cycle. However, a recent study demonstrated that downregulation of Ran in human colon cancer cells provoked S-phase arrest in K-Ras mutated cell lines, but not in cells expressing wild-type K-Ras [22]. We had previously checked for wild-type K-Ras in our cell lines and found no known genetic alterations [14]. However, propidium iodide analysis showed an increase in the sub-G1 phase in our cells treated with shRNA Ran, indicating possible presence of apoptotic cells following Ran depletion, that was not reported in the study of Morgan-Lappe et *al*. [22] on colon cancer cells. This may be explained by the fact that they tested the cell cycle of their cells after 48 h of Ran depletion. At that time point, there was only a slight difference in the sub-G1 cells proportion for TOV112D TetR, while there was no difference in TOV1946 TetR. Nevertheless, the induction of apoptosis in colon cancer cells was observed by a caspase-3 activation assay after 72 h, demonstrating that Ran downregulation leads to a caspase-3 associated apoptosis in both models. The fast induction of apoptosis and the extent of the response of the ovarian cancer cells to Ran depletion suggest a particular sensitivity of ovarian cancer cells to the loss of Ran.

As Ran depletion showed a dramatic effect on proliferation *in vitro*, we were interested in the effect of its downregulation *in vivo*. All TOV112D TetR cell lines formed tumors when injected sub-cutaneously into SCID mice, indicating that the repressor did not impair the capacity of these cells to grow as xenografts in a murine model. When induced, shRNA targeting Ran resulted in a clear delay in tumor outgrowth and was associated with induction of apoptosis within the tumor cells, as observed by a cleaved caspase-3 IHC in the xenografts. Interestingly, this delay was more evident in clones which had the highest level of Ran suppression. These results are in line with a study where siRNA was used to downregulate Ran in a murine neuroblastoma model [23]. This group showed that Ran depletion in mice resulted in reduced tumor growth and was also associated with induction of apoptosis.

It is likely that, *in vivo*, selective pressure results in the outgrowth of cells able to escape Ran depletion. The initial decrease in Ran expression following induction, compared to its high expression in the eventual tumors that formed, support this notion. In the shRNA Ran mixed population, cells expressing less or no shRNA against Ran would have a selective growth advantage and could thus give rise to the eventual tumor. A similar selection probably also occurs in the shRNA Ran clone #3, although this epigenetic based effect results in tumors taking longer to appear. In line with this hypothesis, the immunohistochemistry of tumors six days after induction showed only a slight decrease in Ran expression in the mixed population in contrast to stronger but not complete inhibition seen in tumors derived from the clonal population. Moreover, immunohistochemistry of all tumors at the day of sacrifice showed that Ran expression was similar to the controls, indicating that Ran repression was lost.

Tumors with reduced expression of Ran showed impaired nuclear Ki67 localization. Membranous and cytoplasmic staining of Ki67 has already been described in hyalinizing trabecular adenoma of the thyroid gland, sclerosing haemangioma of the lung, pleomorphic adenoma of the salivary gland and invasive breast carcinoma [24-27]. However, the mechanism of Ki67 localization remains unknown. Given the role of Ran in nucleocytoplasmic transport, it is possible that this protein plays a role in the transport of Ki67.

It has previously been shown that Ran GTPase is involved in the host innate immune response against microbial pathogens [28]. More recently, a study revealed that transgenic mice overexpressing Ran have reduced nuclear accumulation of c-Jun and c-Fos (AP-1 factors) in T cells, leading to diminished cytokine secretion, decreased proliferation, and impaired *in vivo *T cell function [29]. It has also been reported that Ran overexpression in human glioblastoma cells increases their resistance to paclitaxel treatment. In these cells, Ran can inhibit Bcl-2 phosphorylation and thereby suppress paclitaxel-induced apoptosis [30]. In EOC, standard treatment is carboplatine/paclitaxel [31]. Unfortunately, only 60-80% of women treated with these drugs respond to first-line treatment and the majority will relapse [32]. Here, we show that Ran depletion has a major impact on apoptosis of EOC cells, suggesting that Ran could be a key player in the resistance of EOC cells to chemotherapy by avoiding paclitaxel-induced apoptosis.

Ran depletion seems to affect cell viability in a variety of cancer cells. In fact, inhibition of Ran expression in ovarian, colon, breast, renal and nasopharyngeal tumor cells leads to growth inhibition and/or cell death [12,13,20,22]. In 2008, the group of Xia and *al *[12], showed that Ran inhibition with siRNA for 72 h induces aberrant mitotic formation, mitochondrial dysfunctions and apoptosis of tumor cells. However, depletion of Ran expression in normal fibroblasts was well tolerated, and did not induce mitotic defects or loss of cell viability.

## Conclusion

In conclusion, we demonstrate in this study that Ran GTPase is essential for EOC cell survival *in vitro *and *in vivo*. Ran downregulation triggers caspase-3 associated apoptosis and causes a delay in tumor outgrowth. Furthermore, tumor outgrowth is associated with the selection of tumor cells which have escaped Ran depletion. These results suggest that Ran could potentially be a suitable therapeutic target for the treatment of ovarian cancer.

## Methods

### Cell culture and generation of cell lines expressing the tetracycline repressor

Two cell lines derived from aggressive EOC tumors were used to downregulate the expression of Ran. Both the TOV112D and TOV1946 ovarian cancer cell lines have been described [14,15] and are known to express high levels of Ran. Cells were grown as previously described, in OSE complete medium (Wisent, Montreal, Qc) containing 10% FBS, 2.5 μg/mL amphotericin B and 50 μg/mL gentamicin (Gibco, Grand Island, NY), at 37°C and 5% CO_2 _[14,15]. We generated stable cell lines clones expressing the tetracycline repressor (TetR) by transfection of the pcDNA6/TR vector (Invitrogen, cat. No. V1025-20, Carlsbad, CA) with Lipofectamine (Invitrogen, Carlsbad, CA). TOV112D TetR and TOV1946 TetR cell lines clones were grown in OSE complete medium supplemented with blasticidin. The tetracycline repressor was required to prevent basal target gene knockdown.

### shRNA vector construction targeting RAN expression

To knockdown the expression of Ran, we used the BLOCK-IT™ kit from Invitrogen. An oligonucleotide encoding a stem-loop structure targeting Ran was cloned in the pENTR/H1/T0 vector to generate an entry vector. The sequence of the oligonucleotide (5'-caccagaagaatcttcagtactattcgaaaatagtactgaagattcttc-3') was derived from a siRNA oligonucleotide previously shown to target Ran [22]. As a non-specific control, an shRNA targeting LacZ with the sequence: 5'-caccaaatcgctgatttgtgtagtcggagacgactacacaaatcagcga-3' was used. Using the Gateway^® ^Cloning technology (Invitrogen, Carlsbad, CA), an LR recombination (between attL and attR sites) between the entry vector and the pLenti X1 Puro DEST destination vector (694-6; Addgene #17297) [33] was performed to obtain the final expression vector.

### Lentiviral production and transduction

The lentiviruses were produced by co-transfecting the pLenti X1 Puro DEST-shRNA vector and the ViraPower Lentiviral Packaging Mix (Invitrogen, Carlsbad, CA) in the 293FT packaging cell line. The supernatant was collected and concentrated by ultracentrifugation. For infection, 2 × 10^5 ^cells per well were plated in 6-well plates in 2 ml of growth culture medium one day before infection to obtain 50-70% confluence. Infections were performed in the presence of 5 μg/ml polybrene^® ^(Sigma, St. Louis, MO). Media was changed 16 h after the infection and puromycin selection was performed after two days.

### Preparation of RNA and Reverse transcription-PCR

Total RNA was extracted from cells grown to 80% confluence in 100 mm Petri dishes using TRIzol™ reagent (Gibco/BRL, Life Technologies Inc., Grand Island, NY). Cells expressing shRNA were induced three days before RNA extraction with 1 μg/ml tetracycline. The quality of RNA was tested with the RNA 6000 nano LabChip kit using a 2100 bioanalyser (Agilent Technologies, Mississauga, ON). All RNA samples had RIN (RNA integrity number) scores of at least eight.

Synthesis of cDNA was done using the QuantiTect Reverse Transcription Kit for RT-PCR (Reverse transcription polymerase chain reaction) (QIAGEN Inc., Mississauga, ON) according to the manufacturer's protocol, using 1 μg of total RNA and 1 μL of RT primer mix(oligo-dT and random primers). cDNA samples were diluted 1:50 in water for the Q-PCR (Quantitative real-time polymerase chain reaction) reaction. Q-PCR was performed using the Rotor-gene 3000 Real-Time Centrifugal DNA Amplification System (Corbett Research, Montreal Biotech Inc., Montreal, Qc). 5 μL of the sample cDNA was mixed with 1 μL of the primers (10 μM) and 12.5 μl Quantitech™ SYBR Green PCR (QIAGEN Inc., Mississauga, ON) for a final volume of 25 μl Negative controls were done in all experiments and ERK-1 served as the control. Two independent experiments were each done in duplicate. Primer sequences were: Ran 5'-agccccaggtccagttcaaac-3' and 5'atggcacactgggcttggata-3', and ERK-1 5'-gcgctggctcacccctacct-3' and 5'-gccccagggtgcagagatgtc-3'. To measure the relative quantity of gene expression, the Pfaffl analysis method was used [34].

### Antibodies

The following antibodies were used for western blotting and immunohistochemistry analysis: anti-Ran goat polyclonal (sc-1156, Santa Cruz Biotechnology, Santa Cruz, CA), anti-cleaved Caspase-3 (Asp175) rabbit polyclonal (#9661, Cell signaling Technologies, Beverly, MA), anti-Ki67 rabbit polyclonal (RM 9106, NeoMarkers, Montreal, Qc) and anti-ß-actin mouse monoclonal (AC15, Abcam, Cambridge, MA).

### Western blot analysis

Equal amounts of total protein extracts were loaded and electrophoresed on SDS-polyacrylamide gels and transferred onto a nitrocellulose or PVDF membrane. The membranes were blocked with 5% milk and probed with primary antibodies. Dilutions used were 1:500 for Ran and 1:250 for cleaved caspase-3. Each primary antibody was detected with a conjugated secondary antibody-HRP and visualized by the enhanced chemiluminescence (ECL) method. Loading control for the samples was confirmed by reprobing membranes with anti-actin.

### Cellular growth

Cells were induced with 1 μg/ml tetracycline three days prior to plating cells (day 0). On day 0, 2 × 10^5 ^cells were seeded onto 6-well plates. Cells were trypsinized, resuspended in media and counted using a hemacytometer every 24 h for the five following days. Each experiment was performed in duplicate and was repeated three times.

### Mitochondrial membrane potential (ΔΨ_m_)

ΔΨ_m _was measured by loading cells with tetramethylrhodamine ethylester (TMRE), a cell permeable, cationic, nontoxic fluorescent dye that specifically stains live mitochondria. TMRE is accumulated by the mitochondria in proportion to membrane potential [35]. Briefly, cells induced with 1 *μ*g/ml tetracycline for different times were incubated with 1.25 *μ*M TMRE (cat. no 87917, Fluka, St. Louis, MO) for 20 minutes at 37°C. Trypsinized cells were then collected with the supernatant, washed four times with phosphate buffered saline (PBS), resuspended in 500 *μ*l of PBS and analyzed with the FL2 channel of a Coulter EPICS XL-MLC Flow Cytometer.

### Nuclear apoptosis detection

Frequency of hypodiploid cells (sub-G_1_) was quantified by flow cytometry after staining the cells with propidium iodide (PI). Briefly, cells induced with 1 μg/ml tetracycline for different times were collected with the supernatant, washed and fixed with 70% cold ethanol for 1 h at 4°C and stored at -20°C until staining was performed. Cells were incubated 30 minutes at room temperature with 500 *μ*l of PBS containing 250 *μ*g of RNase A (Roche, Mississauga, ON). 50 *μ*g/ml of PI (P-4864, Sigma-Aldrich, St. Louis, MO) was added and incubated for 10 more minutes at room temperature. Cells were analyzed with the FL3 channel of a Coulter EPICS XL-MLC Flow Cytometer.

### Mice and tumor formation

Six weeks old female SCID CB17 mice (Charles River, Montreal, QC) were injected with 1 × 10^6 ^cells suspended in a mix of 1:1 PBS and matrigel (BD Biosciences, Mississauga, ON) at subcutaneous sites. Doxycycline-supplemented food (625 mg/kg) (Harlan, Indianapolis, IN) was given at day 9 in experimental groups and control groups continued to receive normal food. One tumor from each group of seven mice was collected and fixed in formalin on day 0, 3 and 6 of the doxycycline-supplemented food and two tumors per group of mice were collected at the moment of the sacrifice. Data on weight of the mice and dimensions of the tumors were collected twice a week. Animals were housed under sterile conditions during all experimentations and were sacrificed before neoplastic masses reached limit points establishes by the Institutional Committee on Animal Protection (ICAP) according to the Canadian Council on Animal Care.

### Immunohistochemistry analysis

Tumors collected from mice were embedded in paraffin after two days of formalin fixation at 4°C. A tissue microarray (TMA) was constructed from the xenograft blocks. Two punches of 0.6 mm diameter were taken from each tumor. The TMA was cut with a 4 μm thickness, sectioned onto glass slides, and stained by an immunoperoxidase method. Briefly, TMA slides were heated at 60°C for 20 minutes, deparaffinized in xylene and rehydrated in an ethanol gradient. Slides were then submerged in citrate buffer (pH 6.0) and heated under pressure (Cuisinart Pressure Cooker) for 15 minutes to unmask antigens. Following a 3% H_2_O_2 _treatment to eliminate endogenous peroxidase activity, slides were blocked with a protein blocking reagent (DakoCytomation Inc., Mississauga, ON) for 15 minutes at room temperature. Sections were incubated with primary antibodies for 60 minutes at room temperature. Tissues were incubated with secondary antibodies conjugated with horseradish peroxidase (HRP) (Santa-Cruz, Santa Cruz, CA) for 20 minutes at room temperature. Reaction products were developed using diaminobenzidine (Sigma-Aldrich, St. Louis, MO) containing 0.015% H_2_O_2_. Nuclei were counterstained with hematoxylin. Substitution of the primary antibody with PBS served as a negative control. In wild-type TOV112D cells staining for Ran is mainly cytoplasmic, whereas Ki67 and cleaved caspase-3 are exclusively nuclear.

### Statistical analysis

For statistical analysis, Mann-Whitney U-tests were carried out using SPSS software (version 16.0). A P-value < 0.05 was considered statistically significant.

## Competing of interests

The authors declare that they have no competing interests.

## Authors' contributions

VB designed the study, performed all the experiments, analysed the data and prepared a first draft of the manuscript. VO derived the TOV1946 TetR cell line and revised the manuscript. JL performed viral transductions and reviewed the manuscript. PNT, DMP and AMMM supervised and coordinated the study and revised the manuscript. All authors read and approved the final manuscript.

## Supplementary Material

Additional file 1 **Effect of Ran depletion on proliferation**. Growth curves for TOV112D TetR (a) and TOV1946 TetR (b) parental cell lines, mixed populations and clones expressing shRNA Ran or LacZ. Cells were induced with tetracycline three days prior to day 0. Cells were trypsinized and counted every 24 h for five days. Values represent the mean ± SD of duplicate wells from three independent experiments. Mann-Whitney test indicates no significant difference relative to the non-induced parental cell line with p-value < 0.05. Click here for file

Additional file 2 **Tumor growth of mice injected with TOV112D TetR shRNA LacZ**. TOV112D TetR mixed population and clone expressing shRNA LacZ were subcutaneously injected in SCID mice and tumor growth was measured biweekly. All mice developed tumors albeit of varying size and tumor volume. Doxycycline-supplemented food inducing shRNA expression started at day 9. M.P.: Mixed population, #1: clone. Values represent the mean ± SE of groups of 4 mice. Mann-Whitney test indicates no significant difference relative to the group of mice that did not received doxycycline, with a p-value < 0.05. Click here for file

Additional file 3 **Immunohistochemistry analysis of the xenografts in control tumors**. Analysis of the xenografts on day 6 of the doxycycline-supplemented food showing morphological hematoxylin eosin (HE) staining and Ran, cleaved caspase-3, and Ki67 expression. Click here for file
