# Supplementary figures and images for: An essential role for Ran GTPase in epithelial ovarian cancer cell survival
Source: Mol Cancer. 2010 Oct 13;9:272. doi: 10.1186/1476-4598-9-272 (PMC2964620; doi:10.1186/1476-4598-9-272)

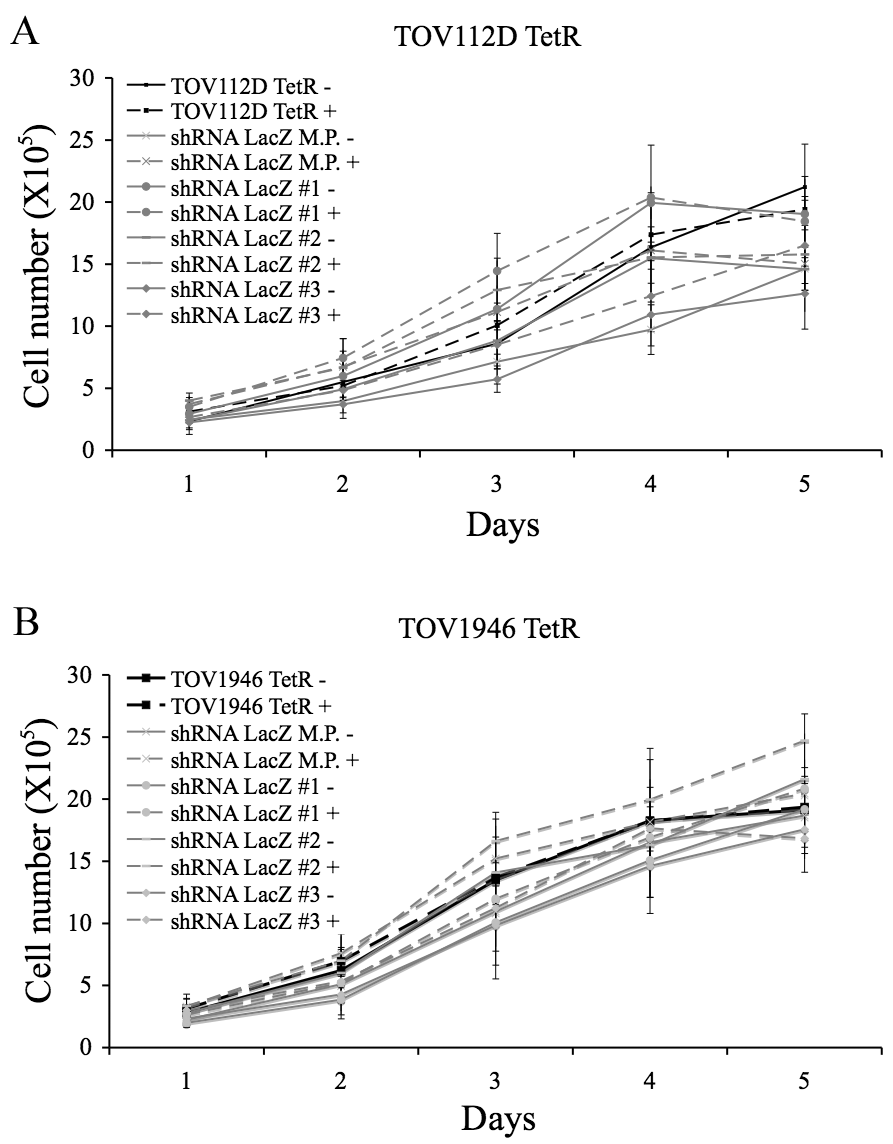

Supplement: Additional file 1 — Effect of Ran depletion on proliferation. Growth curves for TOV112D TetR (a) and TOV1946 TetR (b) parental cell lines, mixed populations and clones expressing shRNA Ran or LacZ. Cells were induced with tetracycline three days prior to day 0. Cells were trypsinized and counted every 24 h for five days. Values represent the mean ± SD of duplicate wells from three independent experiments. Mann-Whitney test indicates no significant difference relative to the non-induced parental cell line with p-value < 0.05. [file 1476-4598-9-272-S1.PNG]

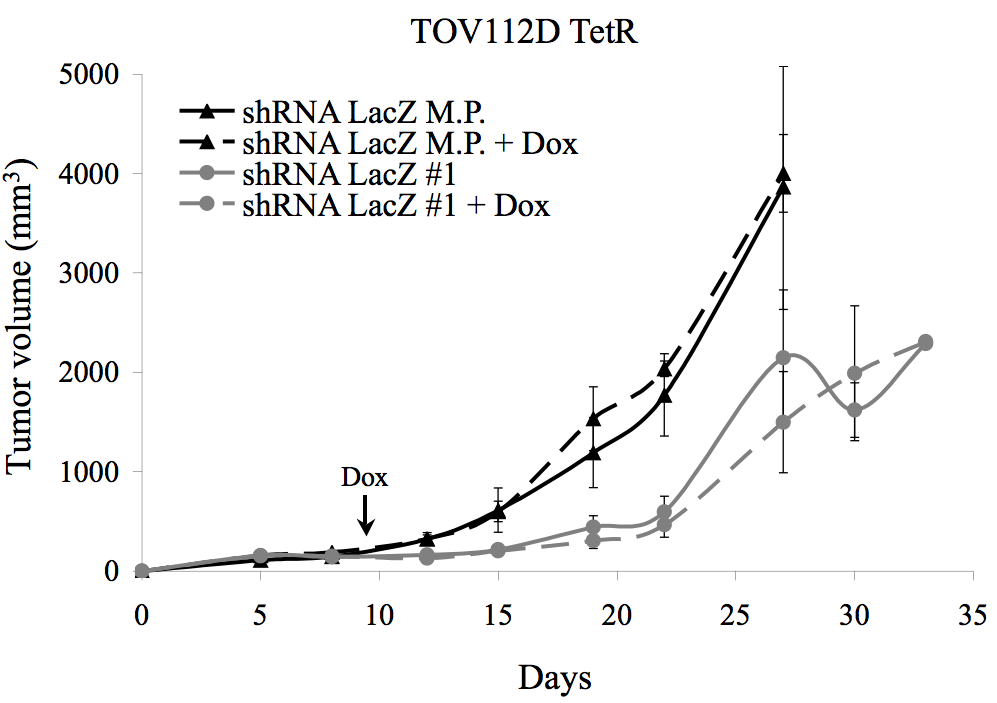

Supplement: Additional file 2 — Tumor growth of mice injected with TOV112D TetR shRNA LacZ. TOV112D TetR mixed population and clone expressing shRNA LacZ were subcutaneously injected in SCID mice and tumor growth was measured biweekly. All mice developed tumors albeit of varying size and tumor volume. Doxycycline-supplemented food inducing shRNA expression started at day 9. M.P.: Mixed population, #1: clone. Values represent the mean ± SE of groups of 4 mice. Mann-Whitney test indicates no significant difference relative to the group of mice that did not received doxycycline, with a p-value < 0.05. [file 1476-4598-9-272-S2.PNG]

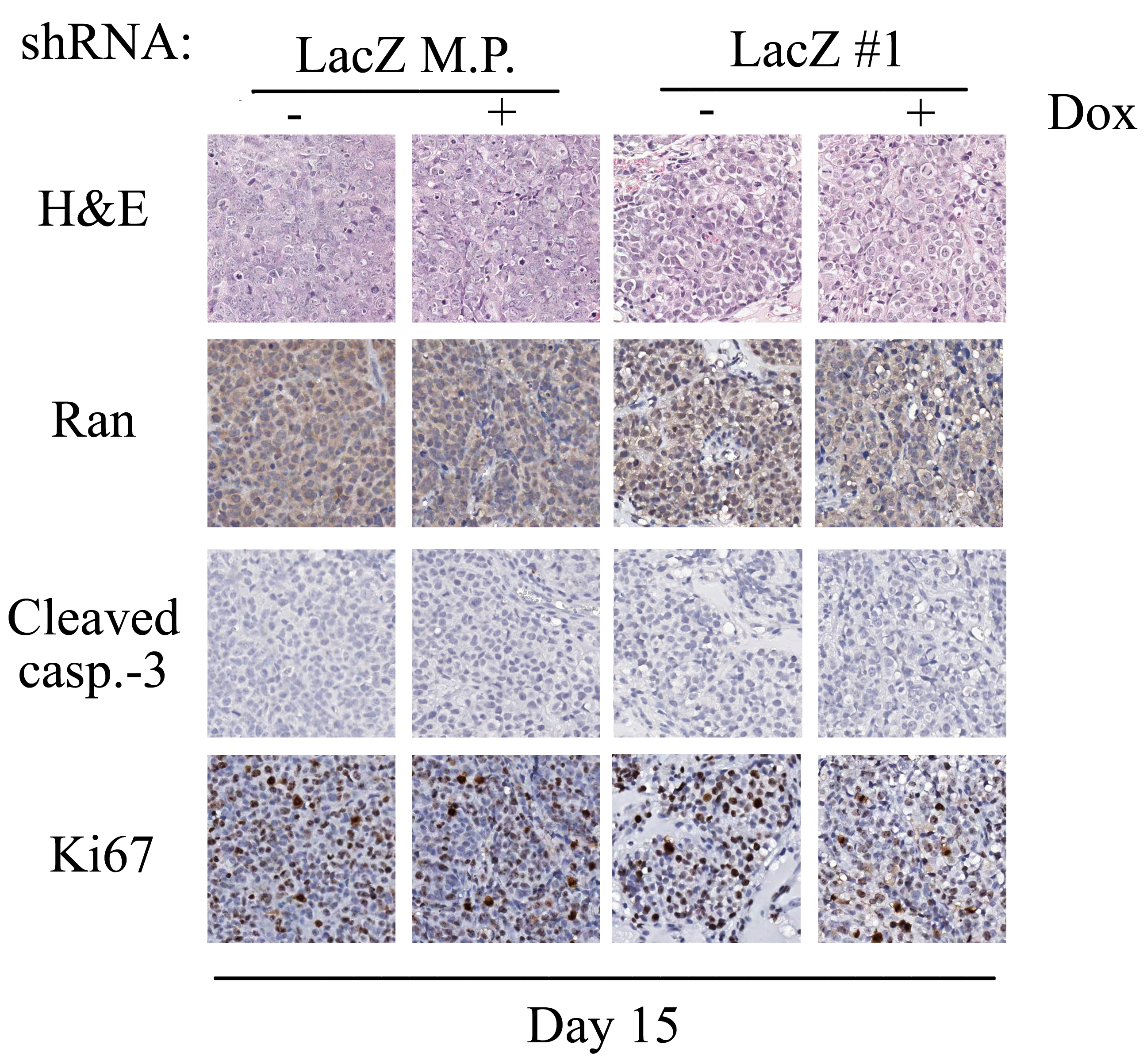

Supplement: Additional file 3 — Immunohistochemistry analysis of the xenografts in control tumors. Analysis of the xenografts on day 6 of the doxycycline-supplemented food showing morphological hematoxylin eosin (HE) staining and Ran, cleaved caspase-3, and Ki67 expression. [file 1476-4598-9-272-S3.PNG]
